# PROTOCOL: Gender transformative approaches in agriculture for women's empowerment: A systematic review

**DOI:** 10.1002/cl2.1265

**Published:** 2022-07-09

**Authors:** Sabina Singh, Ashima Mohan, Ashrita Saran, Ranjitha Puskur, Avni Mishra, Linda Etale, Steven Michael Cole, Edoardo Masset, Hugh Sharma Waddington, Heather MacDonald, Howard White

**Affiliations:** ^1^ Campbell South Asia New Delhi India; ^2^ International Rice Research Institute Manila Philippines; ^3^ International Institute of Tropical Agriculture Dar es Salaam Tanzania; ^4^ London School of Hygiene and Tropical Medicine London United Kingdom; ^5^ Carleton University Ottawa Canada; ^6^ Global Development Network New Delhi India

## Abstract

This is the protocol for a Campbell systematic review. The objective of this systematic review is to assess the effectiveness of interventions with gender transformative approach (GTA) components in improving women's empowerment in low‐ and middle‐income countries, and to curate evidence on the mechanisms through which GTA works to improve women's empowerment in agriculture.

## 1 BACKGROUND

### 1.1 The problem, condition or issue

#### 1.1.1 Gender inequality as a persistent problem

Women constitute 43% of the agricultural labour force in low‐ and middle‐income countries. Their share in the agricultural labour force ranges from almost 20% in Latin America to about 50% in Eastern and South‐Eastern Asia and Sub‐Saharan Africa (Food and Agriculture Organization of the United Nations [FAO], 2011). Although these statistics highlight women's contribution to agriculture to a limited extent, they systematically obscure the issues of unequal access to land, capital, natural resources, and assets among women (Doss, 2014).

Much of the agricultural research and practice so far has been confined to using a women‐in‐development (WID) or gender‐accommodative approach which promotes women's participation within existing institutional and social contexts. The WID approach was centred around addressing women's invisibility in economic contribution and exclusion from development opportunities. Consequently, the agricultural projects at that time focused on women as a category devoid of any social context. The criticism of WID led to Gender and Development (GAD) approach that emphasized mainstreaming gender into the development process with the objective of tackling women subordination and promoting empowerment. The prominent frameworks for this approach popularized the use of sex‐disaggregated data to reflect the inequalities in gender roles, access to assets and control over the same. However, the approach still portrayed women as a category in isolation and devoid of the gender dynamics (Okali, 2012). The gender integration practices, though useful for identifying individual needs of women, have been critiqued for fixing men and women as categories. This homogenization, primarily on men and women's roles and access to resources does not recognize the power relations operating at various levels of society and their interaction (intersection) with individual characteristics such as age, race, ethnicity, social status, and so on (Wong et al., 2019).

This approach, thus, ensures that the symptoms of inequality such as individual access to land, resources and technology are problematized rather than the structural or contextual issues that lead to these inequalities (Okali, 2012). The individualistic approach of addressing gender inequality is problematic as there is every likelihood that contextual issues like established gender norms or institutions may pose barriers to any change that might benefit women (Cole et al., 2014). A relatively new response, that of gender transformative approaches (GTAs), is gaining momentum in recent years in Africa and Asia.^1^

#### 1.1.2 GTAs

The interventions targeting men or women alone may reinforce existing gender norms and accentuate gender gaps. GTA aims to address this by including both men and women as equal partners. GTA aims to encourage ‘critical awareness among men and women of gender roles and norms; promote the position of women; challenge the distribution of resources and allocation of duties between men and women; and/or address the power relationships between women and others in the community, such as service providers or traditional leaders’ (Rottach et al., 2009; p. 8).

What elevates GTA over gender accommodative approaches is that the latter focuses on strategies to ensure participation of women, whereas the former aim to build critical consciousness and address gender norms (Cole et al., 2020). GTA considers women's and men's roles, relations and distribution of resources, and employs a keen understanding of social norms that sanction these power relations. GTA, thus, questions the existing gender roles and norms, and attempts to address the root causes of inequality by identifying harmful norms and replacing them by engaging multiple actors from various scales (including both men and women) at micro, meso and macro levels (Cole et al., 2014). In sum, GTA intends to tackle gender inequalities within a context by transforming structural barriers and in particular factors like norms, attitudes, behaviours and social systems that reinforce gender inequality (AAS, 2012).

Strategies and interventions employing GTA are likely to experience greater challenges in implementation as they strive to address the structural or contextual issues of gender inequality. Despite these challenges, proponents of GTAs argue that they hold the potential for long‐term and sustainable change (Kantor, 2013; Rottach et al., 2009).

### 1.2 The intervention

Various interventions for women's economic empowerment, farmer organizations and governance, climate‐change resilience, nutrition and hygiene, livelihood improvements, savings and microfinance, value‐chains and engaging with youth often use GTAs (FAO, IFAD and WFP, 2020). The interventions using GTAs attempt at raising awareness among men and women about gender norms and roles, elevate women's status by challenging the unequal power relations by questioning the gender roles and resource distribution (Rottach et al., 2009).

An initial screening of literature on GTAs in agriculture was done to identify potentially eligible studies. The same was useful in understanding the components of interventions using GTA. The studies identified and deemed eligible for the review suggest that interventions using communication tools such as drama skits (Cole et al., 2020), community conversations (Mulema et al., 2020) aim at engaging men and women to discuss and examine unequal gender roles and responsibilities have a potential in changing gender perceptions and attitude. The same translates to women's increased access to resources, voice and choice in decision‐making leading to women's empowerment.^2^ Also, interventions that involve elevating women's status within household or communities through transfer of physical assets such as livestock and build social capital through women's self‐help groups or training enhance women's decision‐making as well as encourage joint decision‐making at the level of household (Carnegie et al., 2020). Further, interventions that strengthen women's knowledge and skills through encouraging women to participate along with men in various training or agricultural extension services, capacity‐building workshops may also lead to increase in women's self‐esteem, confidence and ability to participate in income‐generating activities (Benitez et al., 2020).

It thus follows that the interventions could either be exclusively about transforming gender norms or transforming gender norms could be one of the components of the intervention targeted at development goals such as achieving economic empowerment, financial inclusion, or livelihood improvement, to name a few. Thus, transformation of gender norms can either be a means to end or a goal itself as per the nature of the intervention.

GTAs can be largely flexible and adapted as per the context. Also, as per the needs of the program, they could be at the level of individual couples, household (engaging other members of household such as children and/or parent‐in‐laws) or at the level of community. The GTAs are complex and may work at multiple levels simultaneously or in a phased manner, starting from identifying individual members and work across multiple levels in the same project (FAO, IFAD and WFP, 2020).

The target groups could either be newly formed or by selecting the members from existing groups at the village or community level such as self‐help groups, farmers' groups, savings and credit societies such as village savings and loan associations (VSLA) or producer organizations, to name a few. Also, the interventions might be delivered in the same sex‐groups or mixed groups including couples and other household members. Peer‐to‐peer training is one of the models where participants from the community provide training. They might either do it on a voluntary basis as in Individual Household Mentoring or get some incentives. The project staff in certain methodologies like Gender Action Learning System (GALS) also facilitates by providing necessary support (FAO, IFAD and WFP, 2020).

### 1.3 How the intervention might work

The existing theoretical frameworks of women empowerment inadvertently led practitioners and policy makers to focus on designing programs to empower women without taking into account other actors involved in gender relations. GTAs suggest that without addressing the root causes of gender inequality, it is not possible to bring change. Though GTAs also work towards building agency, access and control over resources and improving gender relations, the emphasis is on the process being more inclusive, whereby men and other community actors are also involved.

**Figure 1:**
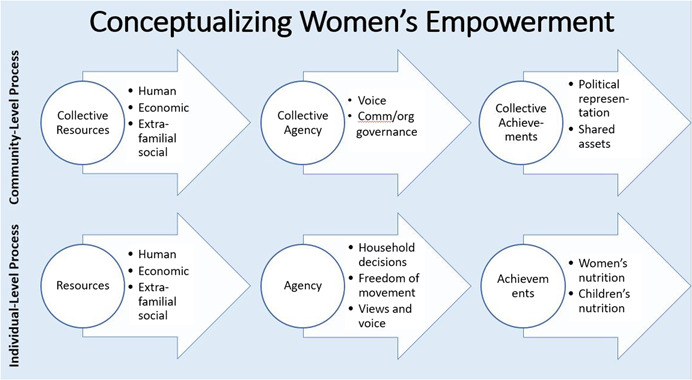
Conceptual framework on women's empowerment. *Source*: https://a4nh.cgiar.org/2017/05/01/a-framework-for-measuring-womens-empowerment-at-multiple-levels/

The inclusive element of GTAs facilitate transformation of gender norms and relations through building collective agency, shared ownership of resources (collective resources) and developmental outcomes (collective achievements)^3^ as shown in Figure 1.

Some of the notable characteristics of GTAs include: an emphasis on transforming social norms, attitudes, and behaviours, not only at the level of individual, households, or communities but also laws, and policies; use of participatory approaches to encourage critical reflection about the social and gender norms and behaviour change at the levels mentioned above; engagement with men, boys and other influencers from the community; and lastly flexibility and adaptability to varied contexts (FAO, IFAD and WFP, 2020).

GTAs in agriculture may be delivered through socio‐behavioural change communication (SBCC) strategies. Some of the participatory approaches and methodologies such as GALS and Gender Household approach are frequently used in agricultural development and food security projects. These methodologies are inherently participatory in nature and differ from one another in their mode of operation as noted below.

GALS, as gender adaptation of Participatory Action Learning System (PALS) sees the connection between unequal gender and social relations, and the development goals, and how gender inequality is a major impediment to achieve development goals. It encourages women and men to identify those barriers using participatory approaches. Participants identify their challenges through visual tools such as drawings. These pictorial tools produced by participants help them identify individual challenges and find solutions themselves (Reemer & Makanza 2014).

At the level of individual, GALS supports individual life planning and strengthens agency as it instils confidence in participants by not only being able to identify their life challenges but also managing those challenges by finding solutions. The participants work towards change at the level of household by addressing unequal gender and power relations. The community component in GALS methodology involves community members through role plays, songs, dramas, and visualizations. The community thus takes over and works towards gender equality, livelihood improvement and policy advocacy (FAO, IFAD and WFP, 2020).

GALS as a GTA thus goes beyond building agency and addresses the root causes of gender inequality by addressing socio‐cultural norms and values that restrict women's access and control over resources.

Similarly, advisory, and agricultural extension services can play a role in transformation of gender norms by involving both men and women as partners in the process. Farnworth and Colverson (2015) discuss the case of Gender transformative Extension and Facilitation System (GT‐EAFS) in Zambia as a system rather than services wherein men and women farmers work together and the system facilitates and promotes production of knowledge by end users.

Another methodology named Gender Household approach targets couples from smallholder coffee farming households who are members of producer organizations. The methodology involves sensitization meeting at the village level, couples' seminars and support from gender staff or facilitator (FAO, IFAD and WFP, 2020).

The interventions involving socio‐behaviour change communication tools encourage discussions among men and women regarding gender roles and relations. Capacity‐building training, workshops, and agricultural extension services that purposively encourage women involvement and participation as well as asset transfer programs might also transform gender norms and relations. The pathways involve raising critical awareness about gender roles and relations, redistribution of access and control over resources, agency and capacity‐building among communities. It is, however, to be noted that the pathway of effect between intervention to outcome is not linear and is mediated by interaction of factors pertaining to individual behaviour and contextual variations.

**Figure 2:**
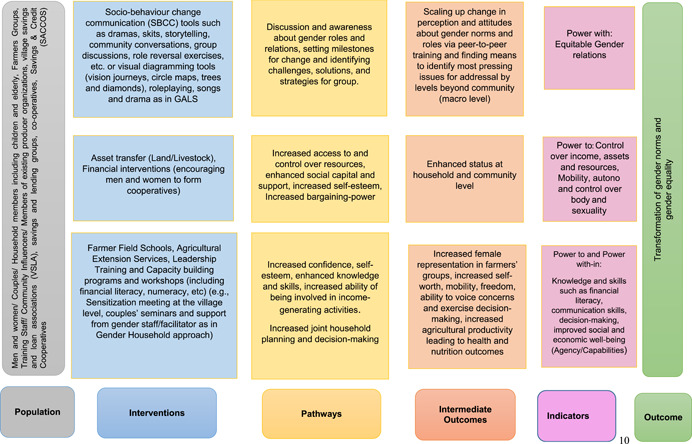
Theory of Change for Gender transformative interventions to increase women's empowerment (based on Benitez et al., 2020; Carnegie et al., 2020; FAO, IFAD and WPF, 2020; Mulema et al. 2020; Yount, 2017).

The theory of change (Figure 2) for this systematic review identifies three broad sets of interventions as: Interventions using socio‐behaviour change communication tools such as visual tools, dramas, storytelling, role reversals, to name a few; Asset transfer and gender household approach.^4^

The communication interventions may encourage participates to engage, discuss and make them aware about gender roles and relations. Specific methodologies like GALS, as discussed earlier, support participants to identify their challenges, life goals and encourage them to find solutions to those problems on their own so that the households realize the constraints of conventional gender norms and roles and challenge those norms and roles to achieve life goals. These discussions are then taken to the level of community groups which may engage in advocacy for change at the level of policy for achieving development goals but in the process transform gender norms and roles since community acknowledges the change.

The second category of interventions mentioned in Figure 2 includes interventions wherein there are incentives to transfer ownership of assets to women or where assets such as animals may be given to households. The interventions encouraging women to open bank accounts or cash‐transfer schemes or encourage both men and women to form co‐operatives for common economic goals. These interventions may enhance women's status within household and communities through ownership and control of resources, increased bargaining power and increased social capital.

The last category of interventions includes agricultural extension services or farmer field schools that encourage members of agricultural households (both men and women) to participate and engage the participants in discussion about gender norms and challenges. The life skills training such as numeracy skills may also be imparted during the training, in addition to extension services. The intervention may lead to increased representation of women in the farmers' groups, enhance their confidence and self‐worth and encourage joint decision‐making. The health and nutrition outcomes resulting from changes in agricultural practice and joint decision‐making may promote that gender transformation is sustainable (Figure 2).

### 1.4 Why it is important to do this review

The issue of gender inequality in agriculture has been an international policy issue for at least three decades. In the year 1983, the State of Food and Agriculture report included for the first time a section on ‘Women in Developing Agriculture’ that acknowledged women's unequal access to land, resources, and technology among other problems of women in agriculture. Their contribution and potential were also noted. The State of Food and Agriculture Report 2010–2011 brought the much‐needed impetus to close the gender gap in agriculture. It emphasized the relationships between gender inequalities, highlighted by international targets at that time under Millennium Development Goal (MDG) 3, and poverty and food security, under MDG 1.

Gender inequality remains a pervasive challenge as the progress on indicators of Sustainable Development Goals (SDG) 5, and other food and agriculture‐related SDG targets, which are far from being achieved by the year 2030 (FAO, 2020). Progress in gender is likely to determine the realization of most of the Sustainable Development Goals (SDGs). Doss et al. (2018) suggest that, in addition to Sustainable Development Goal 5 (SDG) on gender equality and women's rights, at least 11 of the 17 SDGs incorporate indicators and targets related to gender dynamics. The interventions addressing gender inequalities will thus also have implications for achieving these other SDGs. The potential advantage of promoting gender equality in tackling hunger and malnutrition, increasing food security and sustainable agriculture is widely acknowledged (e.g., FAO, 2021). It is imperative that for all round development of society, the social, structural and political dimensions of gender inequality are addressed. Gender equality is also important from a human rights perspective.

The genesis of GTAs traces back to the health sector, and sexual and reproductive health, in particular. A systematic review exists on interventions to shift gender norms (Stewart et al., 2021). A systematic review on farmer field schools incorporated evidence about the effectiveness of approaches to target women farmers (Phillips et al., 2014). But there is no existing systematic evidence about GTA in agriculture more generally. An evidence and gap map^5^ of experimental research on male engagement in gender transformative programming on sexual and reproductive health rights (SRHR) suggests that male engagement in the domain of SRHR with gender transformative programming remains relatively neglected and requires development (Ruane‐McAteer et al., 2019, 2020).

Existing reviews incorporating participatory approaches in agriculture have used mixed‐methods to synthesize evidence (Waddington & White, 2014). A broad review, incorporating questions relating to intervention processes as well as impacts, is therefore likely to be of greatest use to support decision making about GTA (McDougall et al., 2021).

## 2 OBJECTIVES

The objective of this systematic review is to assess the effectiveness of interventions with GTA components in improving women's empowerment in low‐ and middle‐income countries, and to curate evidence on the mechanisms through which GTA works to improve women's empowerment in agriculture.

The following questions have been identified for the review:

1. What are the components of interventions with GTA in agriculture? This refers to the design elements of GTA interventions.
2. What are the effects of GTA in agriculture on women's empowerment, agriculture and nutrition outcomes?
3. What are the barriers to and enablers of the design and implementation of GTA in agriculture to participation and achieving intended outcomes in different contexts?
4. What does the evidence tell on the mechanisms and processes through which GTA operates to empower women in different contexts and at different levels of change, that is, micro, meso and/or macro?

Review question 1 will identify and describe interventions using GTA in agriculture. A thematic synthesis of impact as well as process evaluations will be carried out. Review question 2 will inform about the effectiveness of GTA interventions in agriculture on outcomes relevant for this review. Statistical meta‐analysis will be carried out to address this study question (e.g., Higgins et al., 2019). Review question 3 will identify and summarize the evidence on implementation issues while review question 4 aims to describe the processes and mechanisms underlying GTA interventions that lead to desired outcomes. Review Questions 3 and 4 will be answered using a combination of theory‐based systematic review (White, 2018) and realist‐informed framework synthesis (e.g., Waddington et al., 2019).

## 3 METHODS

### 3.1 Criteria for considering studies for this review

#### 3.1.1 Types of studies

Studies with experimental or quasi‐experimental counterfactual design as well as factual design will be analysed to describe the design components of interventions with GTA in agriculture (review question 1). Studies with experimental or quasi‐experimental counterfactual design will be used to address evidence of effects (review question 2). Eligible designs include those in which the authors use a control or comparison group and in which one of the following is true:

– Individuals and cluster of participants are randomly assigned to intervention groups (using a process of random allocation, such as a random number generation);
– A quasi‐random method of assignment has been used and pretreatment equivalence information is available regarding the nature of the group differences;
– Participants are non‐randomly assigned but are matched by relevant demographic characteristics (using observables, or propensity scores calculated from observables) and/or according to a cut‐off on an ordinal or continuous variable (regression discontinuity design); or
– Participants are non‐randomly assigned, but statistical methods have been used to control for differences between groups (e.g., using multiple regression analysis, including difference‐in‐difference, cross‐sectional (single differences), or instrumental variables regression).

No restriction will be placed on the duration of follow‐up.

Studies analyzing factual evidence on processes will be included to understand enablers and barriers (review question 3). This evidence may use qualitative data collection such as key informant interviews, focus group discussion or other methods of rural appraisal including participatory data generation processes, or mixed‐methods approaches typically found in process evaluations. Review question 4 will be addressed by integrating evidence of effects with evidence on enablers and barriers.

#### 3.1.2 Types of participants

The population of interest for review question 2 is men and women engaged in agriculture in low‐and middle‐income countries, as defined by the World Bank.^6^ They may be of any age, employment or landholding status.

The population of interest for review questions 3 and 4 can include programme staff, community‐level influencers (e.g., youth or elderly) in addition to programme participants and members of producer organizations or village savings and loan associations, as shown in the theory of change (Figure 2).

If the study population includes participants from non‐agriculture sector along with the agriculture and allied sector, we will only include participants from the agriculture and allied sectors given the segregated data is available for such participants.

#### 3.1.3 Types of interventions and comparisons

Eligible interventions are GTAs—that is, strategies or interventions implemented in agriculture to transform gender roles. GTA may be differentiated from other gender integrative approaches in that the end users are involved in identifying and defining the problem as well as in finding its solution. Within these approaches, other actors such as development partners or government and NGO officials may act to enhance the capacities of the community, and/or provide an enabling environment for critical reflection and decision making. Another distinctive feature of GTA is the involvement of men along with women while the gender integrative interventions do not.

The FAO, IFAD and WFP (2020) in their compendium on 15 good practices for food security, improved nutrition and sustainable agriculture describe 15 GTAs as listed in Table 1. This is by far the most exhaustive list of GTAs in the field. Such a list is useful in retrieving literature on GTA in agriculture. However, we are not confining ourselves solely to the approaches mentioned in this list. We will include interventions that aim to impact women's empowerment using gender‐transformative approaches by transforming gender norms and addressing structural barriers within households, communities and markets. GTA may therefore be targeted at micro (intra‐household), meso (community and local markets) and macro (national policy) levels. We will exclude studies that do not explicitly focus on gender‐transformative interventions in agricultural settings. We will include studies where GTAs may constitute one of the approaches in the intervention.

**Table 1:** Types of GTA by level of entry/operation

Entry levels	Gender transformative methodologies
Household and intra‐household level	Gender Action Learning System (GALS) (Oxfam Novib, IFAD, Hivos, Twin and Twin Trading) Gender Household Approach (Hanns R. Neumann Stiftung) Gender Model Family (SEND) Individual Household Mentoring (IFAD) Journeys of Transformation (Promundo) Nurturing Connections© (HKI) Joint Programme to Accelerate Progress towards the Economic Empowerment of Rural Women (JP RWEE) (FAO, IFAD, WFP and UN Women)
Groups and communities levels	Community Conversations (WFP) Dimitra Clubs (FAO) Farmers' Field and Business Schools (FFBS) (CARE) Farmer Field and Life School (FFLS) and Junior FFLS (JFFLS) (FAO) Gender Action Learning System (GALS) (Oxfam Novib, IFAD, Hivos, Twin and Twin Trading) Gender Mainstreaming in Member‐based Organizations (Trias) Social Analysis and Action (SAA) (CARE) Joint Programme to Accelerate Progress towards the Economic Empowerment of Rural Women (JP RWEE) (FAO, IFAD, WFP and UN Women)
Organizations/formal institutions/private sector levels	Gender Action Learning System (GALS) (Oxfam Novib, IFAD, Hivos, Twin and Twin Trading)
	Models to Empower Women in Outgrower Schemes (AgDevCo) Joint Programme to Accelerate Progress towards the Economic Empowerment of Rural Women (JP RWEE) (FAO, IFAD, WFP and UN Women)
Lead agency/Project staff level	Journeys of Transformation (Promundo) Social Analysis and Action (SAA) (CARE)

For impact evaluations, used to answer review question 2, the comparison group may receive usual access to usual agricultural services, a different GTA, or an alternative approach to empowering women (e.g., a gender‐accommodative approach). The control or comparison conditions may therefore include gender accommodative or integrated approaches, GTA implemented with different intensity, or different policies altogether. Broader evidence, used to answer review questions 3 and 4, does not need to use explicit comparison groups.

Any duration or frequency of programme delivery is eligible.

#### 3.1.4 Types of outcome measures

Eligible primary outcomes include measures of gender norms and attitudes, women's participation in activities and decision making and agriculture outcomes. The ultimate outcome is transformation of gender norms and may be accompanied by intermediate outcomes mentioned below, where all other outcomes are primary outcomes except nutrition outcomes. This may however be noted that these outcomes are tentative outcomes based on our theory of change. We will, however, inductively classify the outcome categories based on the eligible studies (Table 2).

**Table 2:** Types of outcomes and example indicators

Outcomes	Indicators
Women's empowerment	Multidimensional empowerment indices such as the Women's empowerment in agriculture index (WEAI) and other ad‐hoc indices built by researchers.
	Agency indicators: decision‐making on use of resources, decisions about agricultural production, access to and decision‐making power about productive resources, control and use of income, Bargaining power, preferences, self‐confidence/self‐worth, aspirations, time‐use outcomes, outcomes associated with ownership and control over land, and other assets, mobility (movement)
	Leadership positions
Agricultural and livelihood outcomes	Awareness among men and women of gendered roles in agriculture and allied activities
	Knowledge and field management outcomes acquired via farmer field schools or rural advisory services or similar interventions
	Access to technology and services, e.g. communication, extension services, other agricultural inputs
	Agricultural yields and incomes.
Socio‐cultural outcomes	Community engagement
	Perception and awareness about gender norms and roles
	Community ownership of assets
Nutrition outcomes	Nutrition (e.g., height‐for‐age of children, body mass index of women and men).

#### 3.1.5 Duration of follow‐up

Any follow‐up duration is eligible. We will code multiple outcomes where studies report multiple follow‐ups.

#### 3.1.6 Types of settings

Interventions implemented in any setting will be included, regardless of location, and type of agriculture (arable, pastoral, aquaculture).

### 3.2 Search methods for identification of studies

The electronic searches of selected databases will be accompanied by grey literature search using organizational websites. Systematic Review databases will also be searched. Hand searches of selected journals will also be done. The visual tool ‘Connected Papers’ will also be used to track related academic research publications.

#### 3.2.1 Electronic searches

We will search the following databases to identify completed and ongoing studies published in English language: AgEcon, US National Agricultural Library (Agricola), World Agricultural Economics and Rural Sociology Abstracts (on CABI), Web of Science Core Collection (1900‐present), Scopus, EconLit (on Ebsco host) EBSCO multifile group of databases, particularly GreenFile and Gender Studies, Geobase (On Engineering Village), PAIS (on Proquest), and EnvironmentIndex (on Ebsco host).

In addition to the above, we will also attempt to access Academic Search Research and Development, Africa‐Wide Information, Business Source Complete, MEDLINE, Embase Classic + Embase, PsycINFO, Database of Abstracts of Reviews of Effects, Health Technology Assessment, and the NHS Economic Evaluation Database, CINAHL (Ebsco platform), Popline.

The search string used for World Agricultural Economics and Rural sociology abstracts (on CABI), is given in the Supporting Information: Appendix 1.

#### 3.2.2 Searching other resources

Campbell Library of Systematic Reviews, 3ie, DFID/FCDO Research for Development (R4D) will be searched. IMMANA grant database, 3ie impact evaluation database and The World Bank IEG evaluations will also be searched.

We will also search the organizational websites and repositories of CGIAR, IDRC, IFAD, IFPRI, AgriProFocus, BMGF, Donor Committee for Enterprise Development, FAO, ILO, International Livestock Research Institute, SNV Netherlands Development Organization, USAID, Swiss Agency for Development and Cooperation, Foreign, Commonwealth and Development Office (FCDO), IPA and J‐PAL, USAID Development Experience Clearinghouse. These websites will be searched for specific keywords such as ‘Gender transformative approaches’ and also by names of various methodologies mentioned in Table 1. The screening of websites for reports and other resources will also be done.

Conference proceedings and papers from the proceedings of the Agriculture, Nutrition and Health Academy conference, the proceedings of the CSAE Conference, the proceedings of the NEUDC Conference and The World Bank Economic Review will also be searched to identify eligible conference papers.

Hand searches/online screening of the table of contents for the last five years for the following journals will also be done:

*Agricultural Systems*
*Agriculture and Human values*
*Asian Journal of Women's Studies*
*Food Policy*
*Frontiers in Sustainable Food Systems*
*Gender and Development*
*Gender and Society*
*Gender, Place and Culture*
*Gender, Technology and Development*
*Global Food Policy*
*Journal of Gender Studies*
*Journal of Gender, Agriculture and Food Security (Agri‐Gender)*
*Journal of Rural Studies*
*Sustainability*
*Women's Studies International Forum*
*World Development*

### 3.3 Data collection and analysis

#### 3.3.1 Description of methods used in primary research

Systematic screening and data extraction will be carried out for searched studies as per the screening and data extraction tools. The details of the procedure are as follows:

#### 3.3.2 Selection of studies

Eppi‐reviewer will be used for data management and data analysis. All the identified studies will be imported to Eppi‐reviewer for screening followed by data extraction. The identified studies will be independently screened by two researchers. The identified records will be first screened at title and abstract as per the screening tool given in the Supporting Information: Appendix B. The screening tool was piloted for screening about 20 studies.

Full‐text screening of the studies included at title and abstract will also be done with reasons by two researchers independently. The disagreements at full text screening will be resolved by discussion and, if necessary, arbitrated by a third reviewer.

#### 3.3.3 Data extraction and management

The data extraction form will be piloted engaging the researchers responsible for data extraction from the Campbell team as well as the content experts. The initial categories for the data extraction will be revised, refined and defined.

The data extraction form at the minimum will have regional and geographical codes, populations, settings, study designs, comparators, codes for interventions and outcomes and their sub‐categories, together with preliminary codes for intervention delivery and implementation. Additional codes related to delivery of intervention and implementation issues will be identified through inductive synthesis and extensive rounds of discussion between the Campbell team and content specialist team. Any codes developed during the synthesis process will be clearly indicated. The intervention description with respect to the design of the eligible studies will be synthesized thematically. A table of intervention methodologies with design components will be given.

Quantitative data for outcome measures such as outcome descriptive information, outcomes means and standard deviations, test statistics (e.g., *t*‐test, *F*‐test, *p*‐values, 95% confidence intervals), as well as sample sizes in each intervention group will also be extracted for studies of effects.

Two researchers will independently extract data and the data extraction reports will be matched for agreements. The disagreements, as at the screening stage, will be resolved by discussion and comparing notes, or through a third reviewer as arbitrator.

#### 3.3.4 Assessment of risk of bias in included studies

Separate tools will be used for assessing the risk of bias or study confidence for quantitative counterfactual evidence and qualitative or mixed‐methods process evidence. The tool to assess risk of bias in randomized and non‐randomized studies will draw on Waddington and Cairncross (2021).^7^

The included randomized and non‐randomized studies will be assessed for confounding, selection bias into the study, attrition, selection bias out of study, departures from intended intervention due to performance bias, departures from intended intervention due to motivation bias, errors in measurement of intervention and outcome, biases in analysis and reporting and unit of analysis error (Waddington & Cairncross, 2021, p. 13).

Appraisal of qualitative process evidence will use the Critical Appraisal Skills Programme (CASP, 2018). Two researchers will independently critically appraise the included studies and compare the assessment with inputs from the arbitrator, if needed, as at the screening and data extraction stages.

#### 3.3.5 Measures of treatment effect

Effect size estimates with 95% confidence intervals will be extracted from included studies. Effect sizes will be measured as mean differences (where studies use the same continuous outcome measured in the same units), standardized mean differences or, in the case of dichotomous outcome variables, odds ratios, together with their standard errors and 95% confidence intervals. The formulae for these effect sizes are presented in other Campbell reviews (e.g., Waddington & Cairncross, 2021).

#### 3.3.6 Unit of analysis issues

Unit of analysis errors will be assessed based on whether the included studies account for clustering of individuals within and across households and other groups such as villages. Standard errors will be calculated to ensure that appropriate clustering by group is done, making adjustments where necessary using standard approaches to estimate the design effect (Higgins et al., 2019; Waddington et al., 2012, p. 21).

#### 3.3.7 Criteria for determination of independent findings

Dependence may occur at the study or intra‐study levels. At the study level, the most complete and latest report, where available, will be selected in case of multiple reports of a single study. However, if different reports discuss different subgroups or outcomes, the data from all these reports will be treated as a single case, using integrative approach (López‐López et al., 2018). At the intra‐study level, only a single effect from each study will be included in each meta‐analysis. Where studies report multiple effects for different outcome types, these will be synthesized separately. Where studies report multiple dependent effects for a particular outcome type (e.g., different measures of empowerment, different follow‐ups, or different participant subgroups), we may use ‘synthetic effects’ to generate a sample‐weighted average before incorporation in meta‐analysis.

#### 3.3.8 Dealing with missing data

Study authors will be contacted if we require additional data that is missing or incomplete. In case of nonavailability/no response from authors, we will report the characteristics of the study but will not include such a study in the meta‐analysis. Where studies do not report group sample sizes to calculate the standard error of the standardized mean difference, the following approximation will be used:

$$se\left(d\right)=\sqrt{\frac{4}{N}+\frac{d^{2}}{2N}},$$

where *se*(*d*) is the standard error of the standardized mean difference, *d* is the standardized mean difference, and *N* is the total sample size.

#### 3.3.9 Assessment of heterogeneity

Heterogeneity will be assessed graphically and statistically. The effect size heterogeneity will be assessed by calculating *I* ^2^ and *τ* ^2^ values. In addition to that, through forest plots, the graphic representation of pooled effect sizes will be given for the key outcome indicators. The causes of heterogeneity, if any, will be identified by visual inspection and moderator analysis. Separate forest plots will be presented for important moderators. Moderators to be considered are shown in Table 3.

**Table 3:** Moderators

Scale	Local (one region of a country)
	Sub‐national (more than one region in country)
	National (entire country)
Setting of intervention	Community
	Household
	Others (specify)
Intervention methodology	GALS
	Gender household approach
	Asset transfer
	Dimitra Clubs
	Any other (specifiy)
Duration of intervention	3 months
	6 months
	1 year
	2 years
	Others (specify
Unit of intervention	Individual (one‐to‐one, including couples)
	Household (may include household members like mother‐in‐law)
	Groups/community such as community‐based groups (like cooperatives)

#### 3.3.10 Assessment of reporting biases

An attempt will be made to search as well as include unpublished studies in this review. However, we will assess the review for publication bias through the funnel plots and Egger's test (Egger et al., 1997).

#### 3.3.11 Data synthesis

Standardized mean differences (SMDs) from continuous outcome variables and odds ratios for dichotomous outcome variables will be synthesized separately. Effect sizes will be pooled statistically using inverse variance weighted random effects meta‐analysis, using the *mean* command in Stata Version 16. Pooled effects will be expressed in metric that is policy‐relevant, for example, a percentage change in odds, or a mean difference measured in natural units of outcome.

#### 3.3.12 Subgroup analysis and investigation of heterogeneity

Heterogeneity will be explored using moderator analysis. Moderator analyses of a single categorical variable will be conducted using a subgroup analysis, analogous to an ANOVA, also under a random‐effects model. Moderator analyses of continuous moderator variables or multiple moderators will be conducted using random‐effects meta‐regression analysis.

#### 3.3.13 Sensitivity analysis

The sensitivity analysis will be done by removing studies from the meta‐analysis one‐by‐one to see if the results of the meta‐analysis are sensitive to any single study. We will also examine sensitivity of findings to risk of bias status (low risk, some concerns and high risk).

#### 3.3.14 Quantitative and narrative synthesis

Also, if the quantitative synthesis is not possible, we will conduct a narrative synthesis. We will report grouping of studies used in synthesis, standardization processes, synthesis methods, criteria for selection of certain studies, methods used to examine heterogeneity. The study characteristics of included studies will be given in a tabular format. Also, the description of the synthesized findings relevant to review questions will be given (Campbell et al., 2020).

#### 3.3.15 Treatment of qualitative research

We plan to do inductive synthesis of eligible studies for qualitative data (review question 3). Qualitative data will be collected from included studies and then grouped using thematic synthesis or content analysis. The data thus synthesized will be presented in a tabular format.

## CONTRIBUTIONS OF AUTHORS

- Content: Sabina Singh, Ranjitha Puskur, Avni Mishra, Linda Etale, Steven Cole, Hugh Sharma Waddington and Howard White are responsible for content. Hugh Sharma Waddington is also the technical lead for the review.
- Systematic review methods: Ashrita Saran and Hugh Sharma Waddington are responsible for systematic review methods.
- Statistical analysis: Hugh Sharma Waddington, Sabina Singh and Ashima Mohan will conduct statistical analysis.
- Qualitative data analysis: Sabina Singh and Ashima Mohan are responsible for performing qualitative data collection and analysis
- Information retrieval: Ashrita Saran is responsible for information retrieval, based on searches designed by Heather MacDonald

## DECLARATIONS OF INTEREST

The authors are not aware of any interests relating to this review. We have not been involved in developing relevant interventions. Edoardo Masset and Howard White have conducted primary research on smallholder agriculture and gender topics. They and Hugh Sharma Waddington have published previous systematic reviews on smallholder agriculture and gender topics. Sabina Singh has conducted primary research on women working as agricultural labourers for her doctoral thesis.

## PRELIMINARY TIMEFRAME

Plan to submit a draft protocol: December 1, 2021.
Plan to submit a draft review: May 1, 2022.

## PLANS FOR UPDATING THIS REVIEW

The review may be updated, subject to availability of funds.

## SOURCES OF SUPPORT

**Internal sources**

• No sources of support provided

**External sources**

This work was carried out under the CGIAR GENDER Platform, which is grateful for the support of CGIAR Trust Fund contributors: http://www.cgiar.org/funders

## Supporting information

Supporting information.

## Footnotes

1
https://www.cgiar.org/innovations/gender-transformative-approaches/ (accessed 23 July 2021).

2 See Underwood and Hendrickson (2014) for a review of communication interventions for gender equality and social equity in aquatic agricultural systems.

3 The use of terms collective agency, collective resources and collective achievements is from Yount's empowerment framework based on Kabeer (1999). https://a4nh.cgiar.org/2017/05/01/a-framework-for-measuring-womens-empowerment-at-multiple-levels/. Accessed on 1 December 2021.

4 There can be varied sets of interventions which may be inherently gender transformative or overlaying gender transformative components to existing interventions. These three sets of interventions are indicative and do not necessarily encompass all interventions that may be gender transformative. The assumptions about causal pathways and intermediate outcomes for each of these are given in the theory of change.

5
http://srhr.org/masculinities/wbincome/

6
https://datahelpdesk.worldbank.org/knowledgebase/articles/906519-world-bank-country-and-lending-groups

7 The risk of bias tool as used by Waddington and Cairncross (2021) may be accessed here.
