# Supplementary material for: PROTOCOL: Gender transformative approaches in agriculture for women's empowerment: A systematic review
Source: Campbell Syst Rev. 2022 Jul 9;18(3):e1265. doi: 10.1002/cl2.1265 (PMC9270661; doi:10.1002/cl2.1265)
Supplement: Supplementary file 1 — Supporting information. [file CL2-18-e1265-s001.docx]

# Appendices

## 1 Search terms used for CAB Abstracts (EBSCO)

( (agricultur* or agrarian or agribusiness* or agronom* or husbandry or farm* or cultivator* or planter* or (rural N3 (enterprise* or produc* or business* or econom*)) or smallhold* or landown* or "land own*" or outgrower*) ) OR AB ( (agricultur* or agrarian or agribusiness* or agronom* or husbandry or farm* or cultivator* or planter* or (rural N3 (enterprise* or produc* or business* or econom*)) or smallhold* or landown* or "land own*" or outgrower*) ) OR SU ( (agricultur* or agrarian or agribusiness* or agronom* or husbandry or farm* or cultivator* or planter* or (rural N3 (enterprise* or produc* or business* or econom*)) or smallhold* or landown* or "land own*" or outgrower*) )AND ( ("Samriddhi Project" OR "Rural Enterprise and Remittance Project" OR "nurturing connections" OR "Journeys of Transformation" OR "Economic Empowerment" OR "Dimitra Clubs" OR ("Community Conversations" N5 gender*) OR (gender* N5 "Household Mentoring") OR "Farmer* Field School*" OR "Farmer* Business School*" OR "Farmer* Field and Life School*" OR "Farmer* Field and Business School*" OR (gender* N9 "Social Analysis and Action") OR SAA OR (Empower* N3 Women) OR "Joint Programme to Accelerate Progress towards the Economic Empowerment of Rural Women" OR GENNOVATE OR (gender* N9 "food system*")) ) OR AB ( ("Samriddhi Project" OR "Rural Enterprise and Remittance Project" OR "nurturing connections" OR "Journeys of Transformation" OR "Economic Empowerment" OR "Dimitra Clubs" OR ("Community Conversations" N5 gender*) OR (gender* N5 "Household Mentoring") OR "Farmer* Field School*" OR "Farmer* Business School*" OR "Farmer* Field and Life School*" OR "Farmer* Field and Business School*" OR (gender* N9 "Social Analysis and Action") OR SAA OR (Empower* N3 Women) OR "Joint Programme to Accelerate Progress towards the Economic Empowerment of Rural Women" OR GENNOVATE OR (gender* N9 "food system*")) ) OR SU ( ("Samriddhi Project" OR "Rural Enterprise and Remittance Project" OR "nurturing connections" OR "Journeys of Transformation" OR "Economic Empowerment" OR "Dimitra Clubs" OR ("Community Conversations" N5 gender*) OR (gender* N5 "Household Mentoring") OR "Farmer* Field School*" OR "Farmer* Business School*" OR "Farmer* Field and Life School*" OR "Farmer* Field and Business School*" OR (gender* N9 "Social Analysis and Action") OR SAA OR (Empower* N3 Women) OR "Joint Programme to Accelerate Progress towards the Economic Empowerment of Rural Women" OR GENNOVATE OR (gender* N9 "food system*")) )AND ( trial or multicenter or "multi center" or multicentre or "multi centre" or nonrandom* or intervention* or effect or effects or impact or controlled or "control group*" or (before N5 after) or (pre N5 post) or ((pretest or "pre test") and (posttest or "post test")) or quasiexperiment* or "quasi experiment*" or "process evaluation" or (program* N3 evaluat*) or "time series" or "time point*" or "repeated measur*" or ((nonequivalent or "non equivalent") N3 control*)) ) OR AB ( trial or multicenter or "multi center" or multicentre or "multi centre" or nonrandom* or intervention* or effect or effects or impact or controlled or "control group*" or (before N5 after) or (pre N5 post) or ((pretest or "pre test") and (posttest or "post test")) or quasiexperiment* or "quasi experiment*" or "process evaluation" or (program* N3 evaluat*) or "time series" or "time point*" or "repeated measur*" or ((nonequivalent or "non equivalent") N3 control*)) ) OR SU ( trial or multicenter or "multi center" or multicentre or "multi centre" or nonrandom* or intervention* or effect or effects or impact or controlled or "control group*" or (before N5 after) or (pre N5 post) or ((pretest or "pre test") and (posttest or "post test")) or quasiexperiment* or "quasi experiment*" or "process evaluation" or (program* N3 evaluat*) or "time series" or "time point*" or "repeated measur*" or ((nonequivalent or "non equivalent") N3 control*)) )

**World Agricultural Economics and Rural Sociology Abstracts (on CABI) Search Strategy (16thFebruary 2022)**

| Sr. No. | Search strings | Number of hits |
| --- | --- | --- |
| 1 | agricultur* or agrarian or agribusiness* or agronom* or husbandry or farm* or cultivat* or planter* or smallhold* or landown* or "land own*" or outgrower* OR livestock OR crop OR crops OR pisciculture OR aquaculture OR breed* OR fish* OR “food produc*” OR floricultur* OR horticultur* OR harvest* OR irrigat* OR "rain water" OR rainwater OR "rain pond*" OR "check dam*" OR forest* OR ("natural resource*" NEAR/1 manage*) OR agroforestry OR agriforestry OR agroecolog* OR agrifood* OR agri-food* OR plantation*) | 9,26,508 |
| 2 | ( (gender* NEAR/3 (transform* OR "household approach*" OR "model famil*" OR mainstreaming OR norms OR role OR roles OR relations)) OR "Rural Enterprise and Remittance Project" OR “Gender action learning system” OR GALS OR "nurturing connections" OR "Journeys of Transformation" OR "Economic Empowerment" OR "Dimitra Clubs" OR ("Community Conversation*" NEAR/5 gender*) OR (gender* NEAR/5 "Household Mentoring") OR "Farmer* Field School*" OR "Farmer* Business School*" OR "Farmer* Field and Life School*" OR "Farmer* Field and Business School*" OR (gender* NEAR/9 ("Social Analysis and Action" OR SAA)) OR (Empower* NEAR/3 Women*) OR GENNOVATE OR (gender* NEAR/9 “food system*”) OR “Financial Education and Entrepreneurial Knowledge” OR (Samriddhi NEAR/3 (project OR mission)) ) | 9,165 |
| 3 | ("impact evaluation" OR "program* evaluation" OR "process evaluation" OR "random* control* trial*" OR "random* trial*" OR rct* OR ( random* NEAR/3 allocat* ) OR "instrumental variable*" OR "synthetic control" OR “intervention stud*” OR (experimental NEAR/1 (study OR design)) OR “quasi experiment*” OR “quasi-experiment*” OR dif-dif OR "double difference" OR difference-in-difference OR "difference in difference" OR "multiple regression" OR "multivariate regression" OR "multivariable regression" OR "bivariate regression" OR "statistical regression" OR "regression discontinuity*" OR “regression analysis” OR "statistical matching*" OR "propensity score matching" OR "covariate matching" OR "coarsened-exact matching" OR "propensity-weighted" OR “mixed method*” OR “cohort stud*” OR “cross sectional” OR “cross-sectional” OR "cohort analysis" OR "quantitative method*" OR "interrupted time series" OR ( before NEAR/5 after ) OR ( pre NEAR/5 post ) OR ( ( pretest OR "pre test" ) AND ( posttest OR "post test" ) ) OR ( "fixed effect*" NEAR/3 ( model OR estimation ) ) OR ( "random effect*" NEAR/3 ( model OR estimation ) ) OR ( ( quantitative OR "comparison group*" OR counterfactual OR "counter factual" OR counter-factual OR experiment* ) NEAR/3 ( design OR study OR analysis)) OR barrier* OR facilitator* OR enabler* OR (("semi-structured" or semistructured or unstructured or informal or "in-depth" or indepth or "face-to-face" or structured or guide) NEAR/2 (interview* or discussion* or questionnaire*)) or “focus group*" or qualitative or ethnograph* or fieldwork or "field work" or "key informant" OR participatory OR “action research” OR “cooperative inquiry” OR “co-operative inquiry” OR case-stud* OR “case stud*” OR “community led” OR community-led OR (bivariate AND model) OR (multivariate AND model) OR “self help group*” OR “self-help group*” OR (program* NEAR/3 (impact* OR assess*)) OR (gender* NEAR/3 (analysis OR approach*)) OR (interview* NEAR/5 theme*) ) | 1,96,085 |
| 4 | (afghanistan OR albania OR algeria OR "american samoa" OR angola OR "antigua and barbuda" OR antigua OR barbuda OR argentina OR armenia OR armenian OR aruba OR azerbaijan OR bahrain OR bangladesh OR barbados OR belarus OR byelarus OR belorussia OR byelorussian OR belize OR "british honduras" OR benin OR dahomey OR bhutan OR bolivia OR "bosnia and herzegovina" OR bosnia OR herzegovina OR botswana OR bechuanaland OR brazil OR brasil OR bulgaria OR "burkina faso" OR "burkina fasso" OR "upper volta" OR burundi OR urundi OR "cabo verde" OR "cape verde" OR cambodia OR kampuchea OR "khmer republic" OR cameroon OR cameron OR cameroun OR "central african republic" OR "ubangi shari" OR chad OR chile OR china OR colombia OR comoros OR "comoro islands" OR "iles comores" OR mayotte OR "democratic republic of the congo" OR "democratic republic congo" OR congo OR zaire OR "costa rica" OR "cote d’ivoire" OR "cote d’ ivoire" OR "cote divoire" OR "cote d ivoire" OR "ivory coast" OR croatia OR cuba OR cyprus OR "czech republic" OR czechoslovakia OR djibouti OR "french somaliland" OR dominica OR "dominican republic" OR ecuador OR egypt OR "united arab republic" OR "el salvador" OR "equatorial guinea" OR "spanish guinea" OR eritrea OR estonia OR eswatini OR swaziland OR ethiopia OR fiji OR gabon OR "gabonese republic" OR gambia OR "georgia (republic)" OR georgian OR ghana OR "gold coast" OR gibraltar OR greece OR grenada OR guam OR guatemala OR guinea OR "guinea bissau" OR guyana OR "british guiana" OR haiti OR hispaniola OR honduras OR hungary OR india OR indonesia OR timor OR iran OR iraq OR "isle of man" OR jamaica OR jordan OR kazakhstan OR kazakh OR kenya OR "democratic people’s republic of korea" OR "republic of korea" OR "north korea" OR "south korea" OR korea OR kosovo OR kyrgyzstan OR kirghizia OR kirgizstan OR "kyrgyz republic" OR kirghiz OR laos OR "lao pdr" OR "lao people's democratic republic" OR latvia OR lebanon OR "lebanese republic" OR lesotho OR basutoland OR liberia OR libya OR "libyan arab jamahiriya" OR lithuania OR macau OR macao OR "macedonia (republic)" OR macedonia OR madagascar OR "malagasy republic" OR malawi OR nyasaland OR malaysia OR "malay federation" OR "malaya federation" OR maldives OR "indian ocean islands" OR "indian ocean" OR mali OR malta OR micronesia OR "federated states of micronesia" OR kiribati OR "marshall islands" OR nauru OR "northern mariana islands" OR palau OR tuvalu OR mauritania OR mauritius OR mexico OR moldova OR moldovian OR mongolia OR montenegro OR morocco OR ifni OR mozambique OR "portuguese east africa" OR myanmar OR burma OR namibia OR nepal OR "netherlands antilles" OR nicaragua OR niger OR nigeria OR oman OR muscat OR pakistan OR panama OR "papua new guinea" OR "new guinea" OR paraguay OR peru OR philippines OR philipines OR phillipines OR phillippines OR poland OR "polish people's republic" OR portugal OR "portuguese republic" OR "puerto rico" OR romania OR russia OR "russian federation" OR ussr OR "soviet union" OR "union of soviet socialist republics" OR rwanda OR ruanda OR samoa OR "pacific islands" OR polynesia OR "samoan islands" OR "navigator island" OR "navigator islands" OR "sao tome and principe" OR "saudi arabia" OR senegal OR serbia OR seychelles OR "sierra leone" OR slovakia OR "slovak republic" OR slovenia OR melanesia OR "solomon island" OR "solomon islands" OR "norfolk island" OR "norfolk islands" OR somalia OR "south africa" OR "south sudan" OR "sri lanka" OR ceylon OR "saint kitts and nevis" OR "st. kitts and nevis" OR "saint lucia" OR "st. lucia" OR "saint vincent and the grenadines" OR "saint vincent" OR "st. vincent" OR grenadines OR sudan OR suriname OR surinam OR "dutch guiana" OR "netherlands guiana" OR syria OR "syrian arab republic" OR tajikistan OR tadjikistan OR tadzhikistan OR tadzhik OR tanzania OR tanganyika OR thailand OR siam OR "timor leste" OR "east timor" OR togo OR "togolese republic" OR tonga OR "trinidad and tobago" OR trinidad OR tobago OR tunisia OR turkey OR "turkey (republic)" OR turkmenistan OR turkmen OR uganda OR ukraine OR uruguay OR uzbekistan OR uzbek OR vanuatu OR "new hebrides" OR venezuela OR vietnam OR "viet nam" OR "middle east" OR "west bank" OR gaza OR palestine OR yemen OR yugoslavia OR zambia OR zimbabwe OR "northern rhodesia" OR "global south" OR "africa south of the sahara" OR "sub-saharan africa" OR "subsaharan africa" OR "africa, central" OR "central africa" OR "africa, northern" OR "north africa" OR "northern africa" OR magreb OR maghrib OR sahara OR "africa, southern" OR "southern africa" OR "africa, eastern" OR "east africa" OR "eastern africa" OR "africa, western" OR "west africa" OR "western africa" OR "west indies" OR "indian ocean islands" OR caribbean OR "central america" OR "latin america" OR "south and central america" OR "south america" OR "asia, central" OR "central asia" OR "asia, northern" OR "north asia" OR "northern asia" OR "asia, southeastern" OR "southeastern asia" OR "south eastern asia" OR "southeast asia" OR "south east asia" OR "asia, western" OR "western asia" OR "europe, eastern" OR "east europe" OR "eastern europe" OR "developing country" OR "developing countries" OR "developing nation*" OR "developing population*" OR "developing world" OR "less developed countr*" OR "less developed nation*" OR "less developed population*" OR "less developed world" OR "lesser developed countr*" OR "lesser developed nation*" OR "lesser developed population*" OR "lesser developed world" OR "under developed countr*" OR "under developed nation*" OR "under developed population*" OR "under developed world" OR "underdeveloped countr*" OR "underdeveloped nation*" OR "underdeveloped population*" OR "underdeveloped world" OR "middle income countr*" OR "middle income nation*" OR "middle income population*" OR "low income countr*" OR "low income nation*" OR "low income population*" OR "lower income countr*" OR "lower income nation*" OR "lower income population*" OR "underserved countr*" OR "underserved nation*" OR "underserved population*" OR "underserved world" OR "under served countr*" OR "under served nation*" OR "under served population*" OR "under served world" OR "deprived countr*" OR "deprived nation*" OR "deprived population*" OR "deprived world" OR "poor countr*" OR "poor nation*" OR "poor population*" OR "poor world" OR "poorer countr*" OR "poorer nation*" OR "poorer population*" OR "poorer world" OR "developing econom*" OR "less developed econom*" OR "lesser developed econom*" OR "under developed econom*" OR "underdeveloped econom*" OR "middle income econom*" OR "low income econom*" OR "lower income econom*" OR "low gdp" OR "low gnp" OR "low gross domestic" OR "low gross national" OR "lower gdp" OR "lower gnp" OR "lower gross domestic" OR "lower gross national" OR lmic OR lmics OR "third world" OR "lami countr*" OR "transitional countr*" OR "emerging econom*" OR "emerging nation*") | 937608 |
| 5 | 1 AND 2 AND 3 AND 4 | 2634 |

## 2 Screening Tool

1. Is the study conducted in Low and Middle Income Countries, as per the latest World Bank Classification?

- Yes, Include and SeeQ.2
- No, Exclude on **country**

1. Does the study target women or men (of any age, employment or landholding status) engaged in agriculture and food systems or engages program staff and community-level influencers (youth/elderly) towards transforming gender norms?

- Yes, Include and See Q. 3
- No, Exclude as **Exclude on population**

1. Does the study evaluate an intervention (policy, programme, project or practice), or a review of evaluations of an intervention aimed at empowering women and changing social norms that govern roles of men and women in agriculture and food systems?

- Yes, Include and See Q. 4
- No, Exclude as **Exclude on intervention**

1. Does the study analyze the effect of the intervention on the empowerment of women and nutrition outcomes?

- Yes, Include and See Q. 5
- No, Exclude as **Exclude on outcome**

1. Does the study has an experimental or non-experimental design with comparison group, instrumental variables and interrupted time series?

- Yes, Include for effectiveness and Stop!
- No, See Q.6

1. Does the study evaluate a program/policy for empowerment of women in agriculture and food systems and discusses implementation issues?

- Yes, Include for process evaluation
- No, Exclude as **Exclude on design**

## 3 Data Extraction Form (Tentative)

- Region
  - East Asia and Pacific
  - Europe and Central Asia
  - Latin America and Caribbean
  - Middle East and North Africa
  - South Asia
  - Sub-Saharan Africa
  - Not Reported
- Country
- Study Design
  - Randomized Controlled Trial
  - Quasi-RCT
  - Discontinuity design
  - Non-randomised study with pre-test and post-test
  - Study with post-test only
  - Factual study design (qualitative or mixed-method process evaluation)
- Population
  - Cultivators
  - Livestock keepers
  - Agricultural labourers
  - Others
- Target group of intervention
  - Women only
  - Men only
  - Both men and women
  - Community influencers
  - Trainers/ Training staff (Supply-side)
- Intervention Categories
  - Community Conversations
  - Financial Interventions
  - Capacity-building interventions
  - Agricultural extension services
  - Individual/Household mentoring or support
- Outcome categories
  - Women’s Empowerment
  - Participation
  - Agricultural and livelihood outcomes
  - Socio-cultural outcomes
  - Nutritional outcomes
- Unit of Intervention
  - Individual (one-to-one, including couples)
  - Household (may include household members like mother-in-law)
  - Group/Community (such as community-based groups)
- Scale of Intervention
  - Sub-Local
  - Local
  - National
- Gender of the target group
  - Women or girls only
  - Men or boys only
  - Both
- Facilitator
  - Internal (Demand-side, from the community, could be volunteer or paid, may have received training from the training staff)
  - External (Supply-side, training staff)
- Nature of work by the Internal facilitator (only if response to above question is Internal facilitator;
  - Voluntary (unpaid)
  - Voluntary (paid)
  - Paid facilitation (not mentioned whether voluntary or not)

Internal (Demand-side)
